# Increasing demand and persistent gaps in perceived need for mental health care: National findings from 2007 to 2021

**DOI:** 10.1177/00048674251393164

**Published:** 2025-12-17

**Authors:** Yuan Tian, Darren Rajit, Frances Shawyer, Ingrid Ozols, Karen Price, Emily Callander, Brett Inder, Sebastian Rosenberg, Vinay Lakra, Ellie Fossey, Graham Meadows, Joanne Enticott

**Affiliations:** 1 Monash Centre for Health Research and Implementation, Faculty of Medicine, Nursing and Health Sciences, Monash University, Melbourne, VIC, Australia; 2 Department of Psychiatry, Faculty of Medicine, Nursing and Health Sciences, Monash University, Melbourne, VIC, Australia; 3 Mental Health at Work, Balwyn North, VIC, Australia; 4 Inaugural Innovation and Enterprise Advisory Board, Faculty of Medicine, Dentistry and Health Sciences, The University of Melbourne, Melbourne, VIC, Australia; 5 School of Public Health and Preventive Medicine, Faculty of Medicine, Nursing and Health Sciences, Monash University, Melbourne, VIC, Australia; 6 Council of General Practice, Australian Medical Association, AMA House, Barton, ACT, Australia; 7 Discipline of Health Services Management, School of Public Health, Faculty of Health, University of Technology Sydney, Sydney, NSW, Australia; 8 Department of Econometrics and Business Statistics, Monash Business School, Monash University, Melbourne, VIC, Australia; 9 Brain and Mind Centre, School of Medical Sciences, Faculty of Medicine and Health, University of Sydney, Sydney, NSW, Australia; 10 Health Research Institute, Faculty of Health, University of Canberra, Bruce, ACT, Australia; 11 Division of Mental Health Services, Northern Health, Melbourne, VIC, Australia; 12 Department of Psychiatry, Melbourne Medical School, The University of Melbourne, Melbourne, VIC, Australia; 13 Department of Occupational Therapy, School of Primary and Allied Health Care, Faculty of Medicine, Nursing and Health Sciences, Monash University, Melbourne, VIC, Australia; 14 Centre for Mental Health and Community Wellbeing, Melbourne School of Population and Global Health, The University of Melbourne, Melbourne, VIC, Australia

**Keywords:** Australia, health service needs, demand for mental health care, health surveys, mental disorders, mental health, national survey

## Abstract

**Aims:**

To examine self-perceived need for mental health care in the Australian adult population between 2007 and 2021.

**Methods:**

The Perceived Need for Care Questionnaire in the 2007 and 2021 National Study of Mental Health and Wellbeing captures types of help needed in five mental health care categories: medication, information, counselling, social interventions and skills training. Needs are rated as unmet, partially met, or fully met. Twelve-month affective, anxiety and substance use disorders are assessed using WHO’s Composite International Diagnostic Interview.

**Results:**

Demand for mental health care (composite of need categories) among adult Australians increased from 14% (2007) to 20% (2021). It also increased in those with common disorders (43.3–58.9%) and without (6.5–9.9%). Highest 2021 demand was in those with affective (76%), followed by anxiety (61%) and substance use (43%) disorders. Rates of demands being fully met remained stable (45% in 2007; 48% in 2021), with rates among those with substance use (24% in 2021), affective or anxiety (38%) disorders, and those without a common disorder (63%). In 2021, needed supports were counselling (16%), information (11%), medication (10%), skills training (6%) and social interventions (5%). Fully met needs were highest for medication (81%), then counselling (57%), information (54%), skills training (41%) and social interventions (15%).

**Conclusions:**

Despite service expansion, less than half of people with demand had fully met needs. Attention is needed on the causes, population-level prevention as well as treatment strategies to address this burgeoning mental health crisis.

## Background and rationale

Mental disorders are one of the leading causes of disability globally, with 970 million people living with a mental disorder worldwide, generating massive economic and health burdens (GBD Mental Disorders Collaborators, 2022; World Health Organization (WHO), 2022). Common mental disorders, including affective, anxiety and substance use disorders, affect a significant portion of the global population (Institute of Health Metrics and Evaluation (IHME), 2024). Addressing these is crucial for reducing the global burden of mental health issues. However, treatment coverage is insufficient globally, with over two-thirds of individuals with mental disorders not receiving the treatment they needed (Evans-Lacko et al., 2018; Moitra et al., 2023; WHO, 2022). This is the case even in well-resourced countries such as Australia, Canada and the United States (Australian Institute of Health and Welfare (AIHW), 2024c; Islam et al., 2022; Moroz et al., 2020; Walker et al., 2015).

The Perceived Need for Care Questionnaire (PNCQ) assesses self-perceived need for mental health care services across five mental health care categories: medication, information, counselling, social interventions and skills training. Originally developed for national surveys in Australia, the PNCQ is used internationally (Meadows et al., 2000b; Prins et al., 2011; Sunderland and Findlay, 2013). For instance, 17% of Canadians aged 15 and older reported a perceived need for mental health care, with one-third of these needs either partially met (21%) or unmet (12%) in 2012 (Sunderland and Findlay, 2013), while the Netherlands Study of Depression and Anxiety found that 75% of Dutch adults aged 18–65 had their needs partially met or unmet (Prins et al., 2011).

Perceived need for care is closely intertwined with service use (Graham et al., 2017; Roberts et al., 2018; Thoits, 2022). Analyses of PNCQ data can build on Andersen’s behavioural model of health service use (Andersen, 1995), linking model concepts such as health literacy, public beliefs of mental health services, barriers to care and need factors. Within need factors, recognizing mental health issues often prompts the acknowledgement of the need for professional care (Jorm, 2012; Meadows et al., 2000a). An individual’s self-assessment of needs can be different from clinical need, the latter determined by clinical judgement and guidelines (Thoits, 2022). Therefore, regularly capturing this information from the population is important for informing health policy.

As measured by the PNCQ, Australia experienced a 3.3% increase in the overall demand for mental health care (composite of need categories) between 1997 and 2007 (Meadows and Bobevski, 2011). Most commonly needed supports from mental health services were information, counselling and skills training (see Table 1 and Supplemental Appendix A for exact definitions) (Meadows and Bobevski, 2011). In 2007, among the 13.8% of adults who perceived a need for care, almost half reported having partially or fully unmet needs (Meadows and Burgess, 2009). Higher proportions reported fully met needs for information (52.2%), counselling (59.0%) and medication (84.2%) (Meadows and Burgess, 2009). To address treatment gaps, the national need or demand for care must be regularly and accurately assessed to prioritize where resources are required most (Wainberg et al., 2017).

**Table 1.** Five types of help needed examined on the Perceived Need for Care Questionnaire (PNCQ).^a^

Preceding phrase: ‘*Considering your mental health care in the past 12* *months, which of the following forms of help did you receive from (the hospitalisations / and / the consultations?)’* or ‘*Do you think you needed this type of help’* Types of help:
1. Information: *‘Information about mental illness, its treatments and available services’*
2. Medication: *‘Medicine or tablets’*
3. Counselling and Psychotherapies:
3.1. *‘Psychotherapy discussion about causes that stem from your past’*
3.2. *‘Cognitive behaviour therapy – learning how to change your thoughts, behaviours and emotions’*
3.3. *‘Counselling – help to talk through your problems’*
4. Social Intervention: *‘Help to sort out housing or money problems’*
5. Skills Training:
5.1. *Help to improve your ability to work, or to use your time in other ways*
5.2. *Help to improve your ability to look after yourself or your home*
5.3. *Help to meet people for support or company*
If any of the types of help are selected from the above list, then: *‘Do you think you got as much of this kind of help as you needed?’*
PNCQ, Perceived Need for Care Questionnaire. *Italics, item text.*

a This table is adapted from Meadows and Burgess’s study (Meadows and Burgess, 2009).

PNCQ, Perceived Need for Care Questionnaire, *Italics*, item text.

In Australia, national surveys conducted in 2007 and 2020–2022 have shown that approximately 1 in 5 people experienced a common mental disorder (affective, anxiety and substance use disorders) in the past year (Australian Bureau of Statistics (ABS), 2007, 2020–2022a). Despite the impact of the COVID-19 pandemic from early 2020, the overall proportion of diagnosed 12-month disorders in the general population remained essentially unchanged from 2007 (ABS, 2007, 2020–2022a), although some shifts in prevalence were observed across age groups and disorder classes (Slade et al., 2025). Nevertheless, established trends indicate a rising demand for mental health care in Australia (Meadows and Bobevski, 2011) including increasing public awareness and mental health literacy (Bonabi et al., 2016; Jorm, 2012), greater use of psychotropic medications (AIHW, 2023; Brett et al., 2017; Meadows et al., 2019a), and policy developments such as the introduction of the Better Access Scheme in 2006, which expanded access to psychological services through Medicare rebates (Department of Health and Aged Care (DoHAC), 2023). In addition, growing economic inequity and cost-of-living pressures have emerged as significant challenges (O’Keeffe, 2024) [see Supplemental Appendix B].

The aims of this study were to report the self-perceived needs for mental health care among the general adult population (aged ⩾ 18) and those with a common disorder (affective, anxiety and substance use disorders) in the past year. Study hypotheses, formulated a priori, are grounded in the above-established trends and policy developments:

1. Consistent with established trends, overall needs (demand) for mental health care in Australian adults has increased between 2007 and 2021.
2. Consistent with established trends, most adults with common disorders in the past year will have their medication-related needs fully met.
3. With the introduction in late 2006 of the Better Access Scheme in Australia, which provides Medicare rebates for psychological treatment delivered by eligible professionals (DoHAC, 2023), the *unmet* needs for counselling services decreased between 2007 and 2021.
4. With the growing economic inequity in the cost of living (O’Keeffe, 2024), there is an increase in *unmet* needs for social intervention (help with finances and housing) between 2007 and 2021.

## Methods

The NSMHW conducted by the Australian Bureau of Statistics (ABS) provides representative population-level information on the prevalence of mental disorders, service use and medication use in Australia. The 2021 NSMHW collected data over an 8-month period from December 2020 to July 2021. It was designed to be broadly comparable to the 2007 NSMHW (ABS, 2020–2022b). Interviews were conducted face-to-face by trained interviewers on a stratified multistage probability sample of the population, aged between 16 and 85 years, living in private dwellings, in urban and rural areas, in all states and territories. Response rates were 60% (*n* = 8841) for the 2007 and 57.1% (n = 5514) for the 2021 waves. For the current study, we focused on the adult population aged 18 years and over. Further survey details are described elsewhere (ABS, 2020–2022b).

Mental and behavioural disorders (here ‘mental disorders’) in the 2007 and 2021 NSMHW were assessed using the WHO’s Composite International Diagnostic Interview, version 3.0 (Kessler and Ustün, 2004). The common 12-month disorders of interest included any affective, anxiety and substance use disorders; these respondents met diagnostic criteria for a lifetime mental health disorder and had symptoms in the preceding 12 months at time of interview.

The PNCQ, developed for the 1997 NSMHW, evaluates mental health care service use within populations (Meadows et al., 2000a, 2000b). It has been adopted in the subsequent 2007 and 2021 NSMHW (Meadows and Burgess, 2009) and other surveys internationally including in Canada (Dezetter et al., 2015; Fleury et al., 2016; Sareen et al., 2007; Sunderland and Findlay, 2013) and the Netherlands (Penninx et al., 2008; Prins et al., 2011). Some extensions of the PNCQ have been made over time, but members of this author group working with the ABS have given close attention to preserving comparability for the main items analysed here.

The PNCQ offers a person-centred approach to capture common types of help participants needed from the five categories in mental health care (Meadows et al., 2000a) (defined in Table 1). In the questionnaire, participants identify the type of help they needed for their mental health in the past year. These are each rated as unmet, partially met or fully met (whereby ‘fully met’ corresponds to receiving all the help they needed; see Supplemental Appendix A). A large proportion of the population reporting *unmet* needs may indicate insufficient access to those supports, while *partially met* needs may also signal insufficient comprehensiveness or quality. The composite of all needs is referred to as ‘demand’ for mental health care while individual need categories pertain to ‘type of help needed’.

Analyses used NSMHW unit-level microdata available from the ABS. The 2007 data was extracted from prior work done by Meadows and Burgess (2009) and 2021 data were analysed within the ABS secure data platform (DataLab). Prevalence was calculated utilizing the Pandas library within Python, incorporating survey weights. Error estimations and confidence intervals were derived with the Jackknife variance estimator, utilizing provided replicate weights as recommended by ABS guidance. Some outputs are suppressed when cell counts are low, complying with ABS disclosure control.

## Results

### Demand for mental health care (2021)

Approximately 3.9 million people perceived some need, representing over 20% of the adult population, see Table 2. Among them, nearly half (48%) reported their demands were fully met, 32.4% partially met and 19.3% reported that none of their demands were met.

**Table 2.** Estimates of mental health care needed in the Australian adult population (2007 and 2021).

**Types of help for perceived needs**		**2007**				**2021**				**2007 & 2021 Demand comparison** ^a^
		People with demand	*Status of needs*			People with demand	*Status of needs*			
			Not met	Partially met	Fully met		Not met	Partially met	Fully met	
Any perceived need **(demand)**	Overall % (95% CI)	13.8 (13.0–14.7)	2.5 (2.0–3.0)	5.1 (4.5–5.8)	6.2 (5.5–6.9)	20.4 (19.0–21.8)	3.9 (3.2–4.7)	6.6 (5.8–7.5)	9.9 (8.7–11.0)	*p* < 0.001
	% of those with demand	100%	18.1%	37.1%	44.9%	100%	19.3%	32.4%	48.3%	
Information	Overall % (95% CI)	7.5 (6.8–8.2)	2.3 (1.9–2.8)	1.3 (1.0–1.6)	3.9 (3.4–4.5)	11.2 (9.9–12.5)	3.4 (2.7–4.0)	1.8 (1.3–2.2)	6.0 (5.1–7.0)	*p* < 0.001
	% of those with need	100%	30.9%	16.9%	52.2%	100%	30.3%	15.7%	54.0%	
Medication	Overall % (95% CI)	7.7 (7.0–8.4)	0.5 (0.3–0.6)	0.7 (0.5–0.9)	6.5 (5.8–7.2)	10.1 (9.0–11.2)	0.9 (0.5–1.3)	1.0 (0.7–1.4)	8.2 (7.3–9.2)	*p* < 0.001
	% of those with need	100%	6.2%	9.6%	84.2%	100%	8.7%	10.0%	81.4%	
Counselling	Overall % (95% CI)	10.6 (9.9–11.3)	2.7 (2.3–3.1)	1.6 (1.3–2.0)	6.2 (5.6–6.9)	16.3 (15.0–17.6)	4.9 (4.0–5.7)	2.1 (1.6–2.6)	9.4 (8.2–10.5)	*p* < 0.001
	% of those with need	100%	25.8%	15.2%	59.0%	100%	29.7%	12.9%	57.4%	
Social intervention	Overall % (95% CI)	4.2 (3.6–4.8)	2.7 (2.2–3.2)	0.4 (0.2–0.6)	1.1 (0.7–1.4)	4.5 (3.7–5.2)	3.6 (2.8–4.3)	0.2 (0.1–0.4)	0.7 (0.4–1.0)	*p* = 0.228
	% of those with need	100%	64.7%	10.2%	25.2%	100%	79.9%	5.4%	14.7%	
Skills training	Overall % (95% CI)	4.0 (3.4–4.6)	1.9 (0.5–2.3)	0.5 (0.3–0.7)	1.6 (1.2–2.0)	5.9 (5.0–6.8)	3.0 (2.4–3.6)	0.5 (0.3–0.8)	2.4 (1.9–2.9)	*p* < 0.001
	% of those with need	100%	47.9%	12.2%	39.9%	100%	50.4%	9.1%	40.5%	

**Figure 1.**
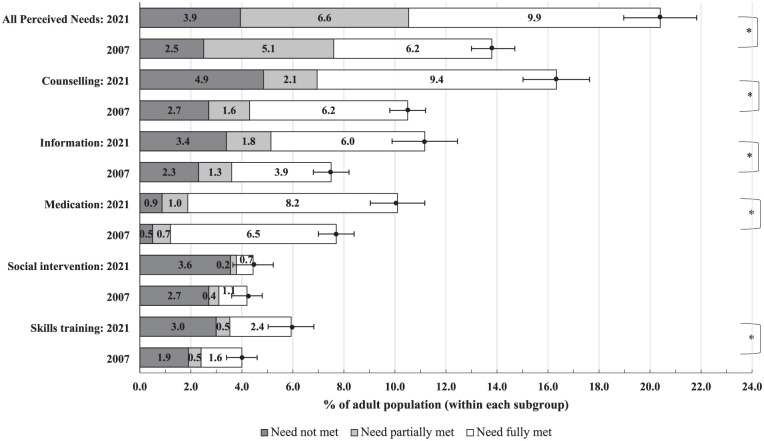
Estimates of type of help needed reported in the Australian adult population (2007 vs 2021). Needs within these categories are classified as unmet, partially met, or met. These three classifications combined apply to individuals with any demand category. A majority of unmet needs may indicate insufficient access, while partially met need may additionally signal insufficient quality. Error bars represent the 95% confidence intervals for the prevalence of any demand. Additional details on these groupings and further confidence intervals are provided in Supplemental Appendix C. *A two-proportion z-test was conducted to compare people with any demand between 2007 and 2021, indicating a statistically significant difference (*p* < 0.001).

CI: confidence interval. Figure 1 shows the visual comparison. Appendix C in Supplementary File has additional details including those reporting no needs for mental health care.

a A two-proportion z-test was used to compare people with any demand between 2007 and 2021.

The population need for counselling was present for 16% or over 3.1 million people. Needs for medication and information was around 10–11%, while social intervention and skills training needs were less common at around 4–6%.

Across the five types of help, the proportion reporting that their need for help was fully met varied: medication needs were the most likely to be fully met (81.4%), followed by counselling (57.4%), information (54.0%), skills training (40.5%) and social interventions (14.7%). This ranking was the same except reversed for those reporting that none of their needs were met: unmet need for medication (8.7%), counselling (29.7%), information (30.3%), skills training (50.4%) and social interventions (79.9%). The proportions reporting that their needs were partially met varied between 5% and 16%: information (15.7%), counselling (12.9%), medication (10.0%), skills training (9.1%) and social interventions (5.4%).

### People with a common disorder (2021)

Overall demand was 9.9% among individuals without a 12-month common disorder, rising to 58.9% among those with a 12-month common disorder (Table 3). Demand was greatest in those with affective (75.9%), followed by anxiety (61.2%) and then substance use (43.1%) disorders.

**Table 3.** Estimates mental health care demand (aggregate of need types) among individuals with and without a 12-month disorder (2021): presence of disorder and broad diagnostic classes.

			People with demand	*Status of demand*			Total^a^
				Not met	Partially met	Fully met	
All adults	No 12-month common disorder	Overall % (95% CI)	9.9 (8.2–11.6)	1.4 (0.9–1.9)	2.2 (1.5–2.9)	6.2 (4.9–7.5)	14,932
		% of those with demand	100.0%	14.4%	22.5%	63.1%	
	With a 12-month common disorder	Overall % (95% CI)	58.9 (55.1–62.7)	13.1 (10.1–16.2)	22.6 (19.8–25.4)	23.1 (19.2–27.0)	4,902
		% of those with demand	100.0%	22.3%	38.4%	39.3%	
Major categories of 12-month mental disorder	Any affective disorder	Overall % (95% CI)	75.9 (70.5–81.3)	16.9 (11.1–22.7)	30.1 (24.4–35.8)	29.0 (23.1–34.9)	1,454
		% of those with demand	100.0%	22.3%	39.6%	38.1%	
	Any substance use disorder	Overall % (95% CI)	43.1 (31.3–54.9)	8.6 (2.8–14.4)	24.4 (13.2–35.6)	10.1 (3.8–16.4)	619
		% of those with demand	100.0%	20.0%	56.5%	23.5%	
	Any anxiety disorder	Overall % (95% CI)	61.2 (56.8–65.6)	13.1 (9.8–16.4)	25.0 (21.4–28.6)	23.1 (18.9–27.3)	3,210
		% of those with demand	100.0%	21.4%	40.8%	37.7%	

**Figure 2.**
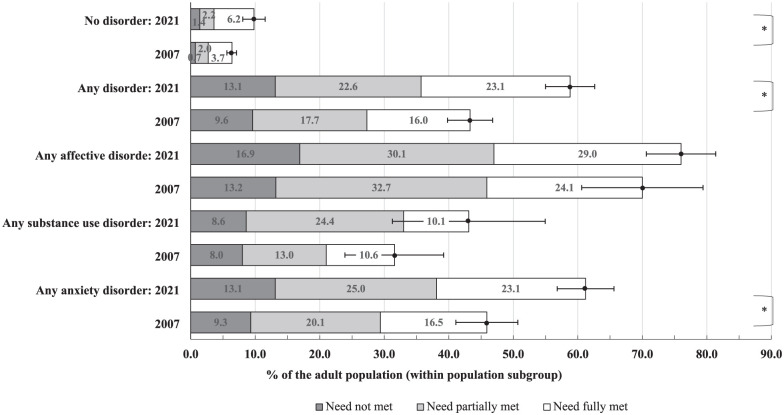
Estimates of type of help needed reported among adult Australians with or without a 12-month common disorder (2007 vs 2021). Needs within these categories are classified as unmet, partially met, or met. These three classifications combined apply to individuals with any demand category. A majority of unmet needs may indicate insufficient access, while partially met need may additionally signal insufficient quality. Error bars represent the 95% confidence intervals for the prevalence of any demand. Additional details on these groupings and confidence intervals are provided in Supplemental Appendix D Table 3. *A two-proportion z-test was conducted to compare people with any demand between 2007 and 2021, indicating a statistically significant difference (*p* < 0.001).

CI: confidence interval.

a Denominator population in question (e.g. 14.9 million adult Australians that had no 12-month common disorder). Figure 2 shows the comparison with 2007. Appendix D in Supplementary File has additional details.

The proportion of individuals reporting that their demands were fully met was 63.1% for those without a disorder, 39.3% for those with affective disorders, 37.7% for anxiety disorders and 23.5% for substance use disorders. Among those without a 12-month disorder, 14.4% had entirely unmet needs, compared w 22.3% among those with a 12-month disorder. These rates were consistent across affective, anxiety and substance use disorders (Table 3). For individuals whose demands were partially met, the percentages were 22.5% for those without a disorder, 39.6% for affective disorders, 40.8% for anxiety disorders and 56.5% for substance use disorders.

Overall, among those both with a common disorder and a demand for mental health care, nearly 8 out of 10 people reported at least partially met needs, but less than half (39.3%) regarded their demand as fully met. Those with substance use disorders reported the least fully met needs (23.5%) and the majority of them reported partially met needs (56.5%).

Table 4 shows the highest needs were reported for counselling by individuals with affective disorders (62.0%), followed by those with anxiety disorders (52.7%). Then, among individuals with affective disorders, 49.3% had a need for information and 48.7% for medications; while in those with anxiety disorders, 37.5% needed information and 32.5% medications. Individuals with substance use disorders reported the lowest demands (e.g. counselling 38.2%, and medication 26.1%).

**Table 4.** Estimates of types of help among individuals with a 12-month disorder (2021).

Types of help for perceived needs (demand)	12-month disorder		People reporting need	Not met	*Status of needs*	
					Partially met	Fully met
Information	Affective disorder	Overall % (95% CI)	49.3 (42.3–56.3)	15.4 (11.4–19.4)	10.2 (5.9–14.5)	23.7 (18.7–28.7)
		% of those with need	100.0%	31.3	20.6	48.1
	Substance use disorder	Overall % (95% CI)	31.8 (20.8–42.8)	14.6 (5.7–23.6)	9.2 (2.5–16.0)	8.0 (3.8–12.2)
		% of those with need	100.0%	45.9	29.0	25.1
	Anxiety disorder	Overall % (95% CI)	37.5 (32.6–42.4)	^a^	^a^	18.3 (14.8–21.8)
		% of those with need	100.0%	^a^	^a^	49.0
Medication	Affective disorder	Overall % (95% CI)	48.7 (41.9–55.5)	5.9 (2.1–9.7)	5.2 (2.5–7.9)	37.6 (30.9–44.3)
		% of those with need	100.0%	12.1	10.6	77.2
	Substance use disorder	Overall % (95% CI)	26.1 (14.8–37.4)	^a^	^a^	19.5 (10.4–28.6)
		% of those with need	100.0%	^a^	^a^	74.6
	Anxiety disorder	Overall % (95% CI)	32.5 (28.8–36.2)	^a^	^a^	25.6 (22.1–29.1)
		% of those with need	100.0%	^a^	^a^	78.8
Counselling/Therapy	Affective disorder	Overall % (95% CI)	62.0 (55.7–68.3)	24.5 (19.2–29.8)	8.6 (5.4–11.8)	28.9 (22.8–35.0)
		% of those with need	100.0%	39.4	13.9	46.7
	Substance use disorder	Overall % (95% CI)	38.2 (27.2–49.2)	17.9 (8.5–27.3)	6.6 (1.3–11.9)	13.7 (7.2–20.2)
		% of those with need	100.0%	46.8	17.4	35.9
	Anxiety disorder	Overall % (95% CI)	52.7 (47.6–57.8)	^a^	^a^	26.5 (22.2–30.8)
		% of those with need	100.0%	^a^	^a^	50.3
Social intervention (housing/money)	Affective disorder	Overall % (95% CI)	22.0 (16.1–27.9)	^a^	^a^	3.7 (1.3–6.1)
		% of those with need	100.0%	^a^	^a^	17.0
	Substance use disorder	Overall % (95% CI)	15.1 (6.8–23.4)	^a^	^a^	^a^
		% of those with need	100.0%	^a^	^a^	^a^
	Anxiety disorder	Overall % (95% CI)	17.3 (13.5–21.1)	^a^	^a^	2.0 (0.7–3.4)
		% of those with need	100.0%	^a^	^a^	11.40
Skills Training	Affective disorder	Overall % (95% CI)	28.0 (22.1–33.9)	14.7 (9.8–19.6)	2.7 (0.7–4.7)	10.7 (6.9–14.5)
		% of those with need	100.0%	52.3	9.5	38.2
	Substance use disorder	Overall % (95% CI)	16.9 (8.9–24.9)	^a^	^a^	^a^
		% of those with need	100.0%	^a^	^a^	^a^
	Anxiety disorder	Overall % (95% CI)	20.2 (16.5–24.0)	^a^	^a^	5.3 (3.5–7.1)
		% of those with need	100.0%	^a^	^a^	26.1

CI: confidence interval.

a Censored due to low cell counts.

Medication-related needs were fully met in the majority of people with affective, anxiety and substance use disorders (75–79%). Less than half of people with affective and anxiety disorders reported fully met needs for information; dropping to only 25% in those with substance use disorders. Counselling-related needs were reported to be fully met in about half of those with affective and anxiety disorders; dropping to 36% in those with substance use disorders. A similar pattern was seen for skill-related perceived needs, as about half of those with affective and anxiety disorders reported fully met needs; dropping to 36% in those with substance use disorders. Social intervention (housing/money)-related needs had the lowest rates for met needs, as 17% in those with affective disorders and 11% with anxiety disorders reported fully met needs.

### General adult population 2007 and 2021 comparison

Figure 1 shows the 2007 and 2021 comparison for the PNCQ. Overall, 14% of the adult population reported a demand for mental health care in 2007, a figure that increased to over 20% by 2021. This was a 43% increase in demand, from 2.2 million individuals in 2007 to 3.9 million in 2021. The proportion of individuals reporting their demands as fully met remained relatively steady, with 45% in 2007 and 48% in 2021. Although the proportion was steady, the growth in demand meant that the number of adults with some degree of unmet (partial or unmet) demand grew from around 1 million in 2007 to over 2 million in 2021.

The type of help that demonstrated the most pronounced growth between 2007 and 2021 was the need for counselling, increasing from 10.6% (95% CI: 9.9–11.3) to 16.3% (95% CI: 15.0–17.6). This was followed by an increase in the need for information, rising from 7.5% (95% CI: 6.8–8.2) to 11.2% (95% CI: 9.9–12.5); for medication, which grew from 7.7% (95% CI: 7.0–8.4) to 10.1% (95% CI: 9.0–11.2) and for skills training from 4.0% (95% CI: 3.4–4.6) to 5.9% (95% CI: 5.0–6.8), whereas the need for social intervention remained relatively steady at 4.2% (95% CI: 3.6–4.8) in 2007 and 4.5% (95% CI: 3.7–5.2) in 2021. See Supplemental Appendix C for details.

### People with a common mental health disorder 2007 and 2021 comparison

In the population without a 12-month disorder, overall demand was 6.5% (95% CI: 5.7–7.2) in 2007, increasing to 9.9% (95% CI: 8.2–11.6) in 2021. At both time points, the majority of these people (approximately 60%) regarded their need for care as fully met. See Figure 2 and Supplemental Appendix D.

Among people with a 12-month disorder, overall demand was 43% in 2007, increasing to 59% in 2021. At both time points, nearly 8 out of 10 people had their perceived need at least partially met, but less than half (37–39%) regarded their need for care as fully met.

Demand is most common in people with affective disorders (70% in 2007; 76% in 2021), compared to those with anxiety (46% in 2007; 61% in 2021) and substance use disorders (32% in 2007; 43% in 2021). For affective disorders, anxiety disorders and substance use disorders, in each group, the needs were fully met in approximately one-third of cases.

## Discussion

In 2021, over 20% of the adult population (3.9 million Australians) self-reported needing mental health care, compared to 14% in 2007 (43% increase). Demand rose among people without a common disorder (6.5% in 2007 to 9.9% in 2021) and those with a common disorder (43.3% in 2007; 58.9% in 2021). While both demand and supply have grown (AIHW, 2025b; Looi et al., 2022), the proportion of people reporting their demands as fully met remained relatively steady, with 45% in 2007 and 48% in 2021. Although proportions were steady, demand growth meant more people having some degree of unmet demand (treatment gap) from 1.2 million (of 2.2 million) in 2007 to over 2 million (of 3.9 million) in 2021. Increasing demand and widening treatment gap were evident in all five types of help categories (medication, counselling, information, social interventions and skills training), supporting our first hypothesis.

These findings are based on data collected primarily during the first half of 2021, aligning with the start of the COVID-19 pandemic. During this period, demand for mental health care reached a peak, as reflected in other national indicators such as Medicare-funded mental health service utilization and calls to crisis services like Lifeline (Enticott and Lakra, 2025; Looi et al., 2022, 2025; Meehan et al., 2025; Yeatman et al., 2023). Notably, this peak in demand occurred even though the overall prevalence of mental disorders remained relatively stable at approximately one in five people (ABS, 2007, 2020–2022a). It is likely that mental health service demand decreased in 2022, following the acute phase of the global crisis. Estimates from other national data sources, such as Medicare mental health services, indicated a 3–5% decline (AIHW, 2025b). If we apply a 5% decline to our 2021 composite demand of 20% yields a projected estimate for 2022 demand of 19%. Despite this slight reduction, the key insights derived from the 2021 findings remain highly relevant and continue to inform our understanding of mental health service needs during and after the pandemic.

### Types of help needed

Population need for medication rose from 8% in 2007 to 10% in 2021. The majority of people needing medication reported that this need was fulfilled (81%), leaving only 19% with unmet need. This finding demonstrates strong accessibility through Australia’s Pharmaceutical Benefits Scheme (PBS), although it is noted that almost 1 in 5 still reported having access issues. Overall, our second hypothesis is supported.

Population need for counselling services increased from 11% in 2007 to 16% in 2021. The introduction of the Better Access Scheme in late 2006 was expected to reduce treatment gaps, but despite expanded psychological services over the past 15 years, an increase in *unmet* demand occurred (from 26% in 2007 to 30% in 2021). Partially met demand decreased slightly from 15% to 13% but so did *fully met* demand from 59% to 57%. Service costs (out-of-pocket fees) and access issues (ABS, 2023–2024; Bartram and Stewart, 2019; Meadows et al., 2019a; Rosenberg et al., 2022) are possible contributors to this finding. This means our third hypothesis is unsupported.

Population need for information increased from 8% in 2007 to 11% in 2021. In people needing information, just over half (54%) reported that this need was fulfilled. This means that almost 1 in 2 people lack all the information they need about their mental health condition, services and treatment options. This large gap for information is surprising given Australia’s mental health literacy initiatives; however, the complexity of the mental health care system and lack of coordinated services are likely to contribute to this problem (Productivity Commission, 2024).

Population need for social interventions remained steady at 4.2% in 2007 compared to 4.5% in 2021. Notably, the majority of people needing this help reported not receiving it (80% in 2021), which had increased from 65% in 2007. This finding supports our fourth hypothesis. Mental health practitioners may not consider financial and housing issues within their scope of care and may be unfamiliar with referral pathways.

Population need for skills training had a 41% fulfilment rate, with 50% reporting not receiving this type of help. These deficiencies are concerning, as such support helps individuals build resilience and reduce future mental health risks (WHO and Calouste Gulbenkian Foundation, 2014). Together, needs for social interventions and skills training broadly fall into psychosocial supports, and these population-level findings are consistent with the estimated 500,000 people with moderate–severe mental illness who required psychosocial support but were not receiving psychosocial support through government-funded programmes in 2022–23 (DoHAC, 2024).

### Those with and without common disorders in the past year

In 2021, overall demand was highest among people with affective (76%), followed by anxiety (61%) and substance use (43%) disorders. The proportions in these groups reporting their demands as fully met were relatively low: 38% for those with affective or anxiety disorders; and, 24% with substance use disorders (24%).

In terms of population numbers, in those reporting a need for mental health care, the majority who report *fully met* needs are Australians without a common disorder (900,000/1,500,000 people), followed by 700,000 (of 2 million) with anxiety, 400,000 (of 1.1 million) with affective and 63,000 (of 260,000) with substance use disorders. Of those who report having fully or partially met needs (i.e. care access), the majority are those 1,400,000 (of 2 million) with anxiety, followed by 1,300,000 (of 1,500,000) without a common disorder, 900,000 (of 1.1 million) with affective and 213,000 (of 260,000) with substance use disorders. Thus, in 2021, Australians without a common disorder are one of the biggest groups accessing care and achieving the best outcomes in terms of self-reported fully met needs. This prompts an evidence-informed question about whether many in this cohort needs to access this (clinical) care or could their needs be fulfilled by other means?

People with substance use disorders were identified as the group with the biggest unmet or unfulfilled demands, and this coincides with other evidence that shows that many in this cohort (19%) ended psychological treatment prematurely (AIHW, 2024a). This raises questions about whether the right treatment at the right time is actually available for this fragile cohort, or is something else required? These considerations are important if Australia plans to address the consistently reported low treatment coverage for those with substance use disorders (Berends and Lubman, 2013; Morley et al., 2016).

### Limitations

Nationally representative surveys, while valuable, have several limitations. These include self-report and recall biases, non-response bias and underrepresentation of marginalized groups such as Indigenous peoples or homeless. Cross-sectional surveys also limit causal inferences. Limited question scope can constrain nuanced understanding.

In addition, the NSMHW criteria capture only the three most common mental disorder groups (ABS, 2020–2022a; Kessler and Ustün, 2004), excluding lower-prevalence conditions such as psychotic or eating disorders. Moreover, individuals with a lifetime history of a common disorder but without symptoms in the past 12 months were not described in our analyses. These limitations are real, but may not substantially affect the population-level results because the three highest prevalent disorders tend to reflect the overall population prevalence due to their large magnitudes. Furthermore, concentrating on those with symptoms in the past year ensures these population figures remain relevant to those who require care the most (Dawadi et al., 2024).

### Recommendations

The increase in demand for mental health care among those who don’t meet criteria for a clinical diagnosis suggests a potential shift in mental health care focus since 2007. In people without a common disorder who reported fully met demands for care, it had previously been reported as effective prevention or maintenance care (Meadows and Burgess, 2009). However, the expansion in this part of the demand population profile between 2007 and 2021 will have other underlying causes. Increased awareness of mental health conditions including adult attention deficit hyperactivity disorder and expanded prescribing practices may explain some of this trend (Brett et al., 2017; Jorm et al., 2006). Despite services growth, there has been no relative increase in the proportion of demand met among those with common disorders. This suggests that resources are expanding to cover a broader population rather than improving care for those with common disorders. Whether this shift benefits population mental health is complex and unclear. As more Australians seek mental health care, the challenge of quaternary prevention (avoiding harm from excessive or inappropriate treatment) also becomes relevant (Meadows et al., 2019a; Porta, 2016).

Mental health is subject to significant resource constraints (Dawadi et al., 2024; Productivity Commission, 2020). When combined with alcohol and drug issues, mental illness now accounts for around 15% of the total burden of disease in Australia (AIHW, 2024b), yet garners less than 7.5% of total health spending, a figure unchanged since 1992–1993 (AIHW, 2025a). In addition, the treatment prevalence paradox is apparent (as Australian mental health care expenditure per person has increased in real terms through two decades by nearly 100%, and by 30% in the last decade) (Meadows et al., 2019b). It becomes clear that priority research is critically needed to identify what really works, for whom, and at the right time, to guide resource allocation and ensure investment in services that deliver the greatest impact and reduce inequities in mental health outcomes (Dawadi et al., 2024).

To meet growing mental health needs, evidence-informed and coordinated strategies are required. Evidence from national surveys, such as those reported herein, should guide these initiatives. Key messages from this national survey analysis include the success of the PBS model, which effectively reduces gap fees (patient co-payments), making medications accessible. However, many psychological services incur a large gap fee, resulting in many deferring care due to cost (ABS, 2023–2024). Improving access to psychological treatments for those with mental disorders is essential. Enhancing primary care pathways and recognizing that primary care not only serves as a referral source but also provides mental health care. Services for grief counselling, relationship counselling, etc. could be useful for those who do not have underlying mental health disorder. Enhancing information accessibility is another critical recommendation. Ensuring clearer, more centralized information on mental health conditions and services could help close the information gap, enabling Australians to better understand how mental illness affects their lives and increasing their chances of connecting to the services they require. Initial assessment and referral mechanisms must be comprehensive, clearly communicated and nationally coordinated. Recommendations from expert working groups provide important advice (Australian Health Ministers’ Advisory Council, 2013; Productivity Commission, 2022).

Furthermore, addressing the low rates of social intervention and skills training requires more recognition of the psycho-social-economic drivers of mental health issues. More support from peer-support workers and allied health professionals is needed. Acquiring these skills and supports provides individuals with more resources to handle stressors in the future. However, Australia’s current limited expenditure on psychosocial services in mental health has been noted (Rosenberg and Harvey, 2021). Finally, there is a need for greater awareness and action related to the broader determinants of mental health including the role of economic, housing and other policy settings in building population ‘mental wealth’ (Occhipinti et al., 2021, 2024; WHO, 2024; WHO and Calouste Gulbenkian Foundation, 2014).

## Conclusion

In mental health care, Australia is displaying the ‘Red Queen’ effect (Barnett and Hansen, 1996; Tiwana, 2014), providing more services yet failing to close the treatment gap. There was a substantial increase in demand for mental health care on a national scale between 2007 and 2021. Despite some service expansion, nearly half of overall demand remains unmet, highlighting a consistent disparity between demand and supply in a highly developed and affluent country. Attention is needed on the causes, population-level prevention and treatment strategies to address the burgeoning mental health crisis. Renewed commitments from federal and state governments must prioritize evidence-informed initiatives that provide targeted interventions for those with the greatest need (Dawadi et al., 2024), while at the same time giving new impetus to opportunities for population-level prevention and earlier intervention. Evidence must come from national representative sources, and from the community members themselves, such as reported herein, ensuring that mental health care delivery leads to real improvements rather than just more services.

## Supplemental Material

sj-docx-1-anp-10.1177_00048674251393164 – Supplemental material for Increasing demand and persistent gaps in perceived need for mental health care: National findings from 2007 to 2021 Supplemental material, sj-docx-1-anp-10.1177_00048674251393164 for Increasing demand and persistent gaps in perceived need for mental health care: National findings from 2007 to 2021 by Yuan Tian, Darren Rajit, Frances Shawyer, Ingrid Ozols, Karen Price, Emily Callander, Brett Inder, Sebastian Rosenberg, Vinay Lakra, Ellie Fossey, Graham Meadows and Joanne Enticott in Australian & New Zealand Journal of Psychiatry
