# Supplementary material for: Increasing demand and persistent gaps in perceived need for mental health care: National findings from 2007 to 2021
Source: Aust N Z J Psychiatry. 2025 Dec 17;60(2):171–83. doi: 10.1177/00048674251393164 (PMC12831812; doi:10.1177/00048674251393164)
Supplement: sj-docx-1-anp-10.1177_00048674251393164 – Supplemental material for Increasing demand and persistent gaps in perceived need for mental health care: National findings from 2007 to 2021 [file sj-docx-1-anp-10.1177_00048674251393164.docx]

# Appendix A. Flow structure of the Perceived Need for Care Questionnaire (PNCQ) instrument. World health organization composite international diagnostic interview (CIDI). When performing a computer-assisted personal interview (CAPI), the interviewer takes a laptop computer to the interview and codes the data into the computer as it is provided.


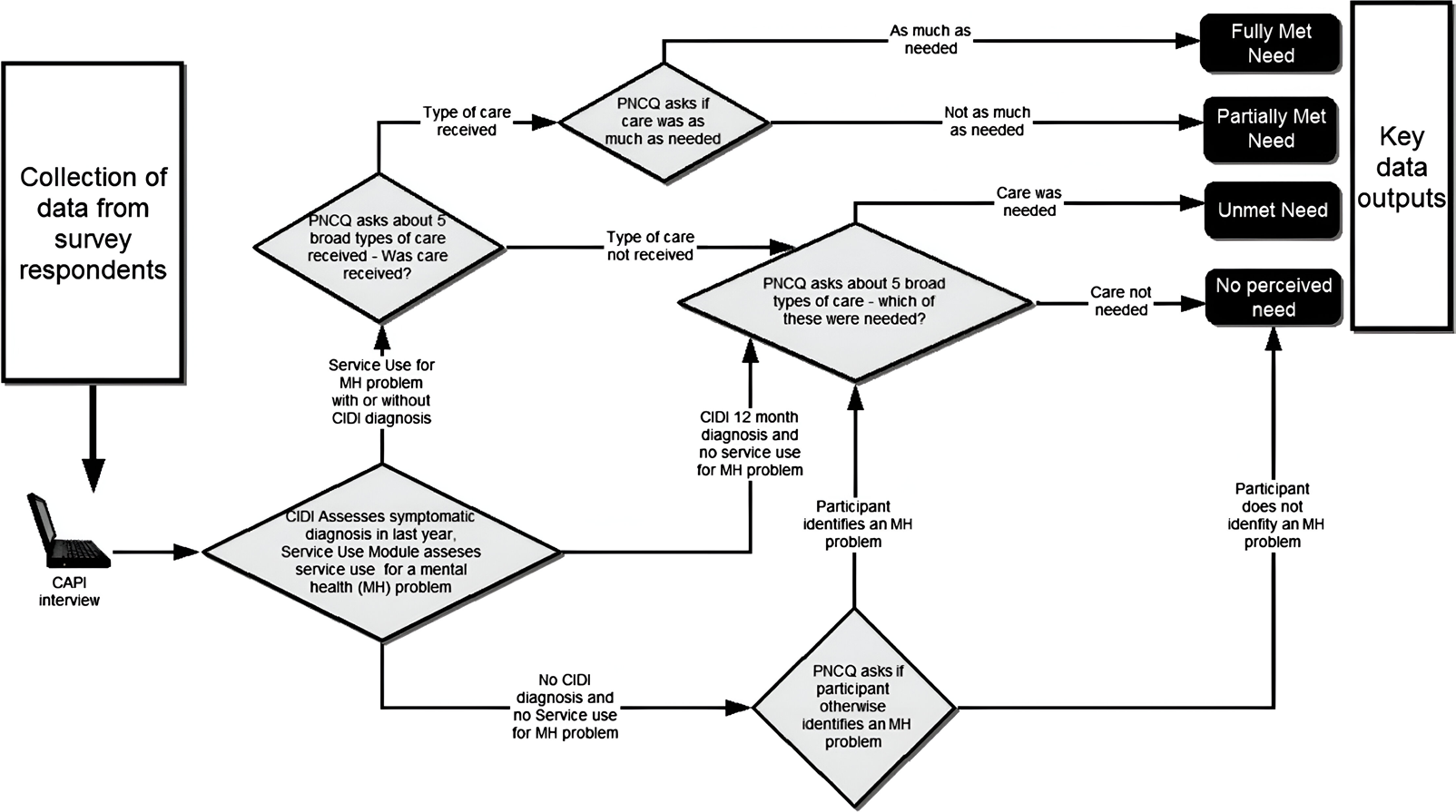


# Appendix B. Details on established trends and policy developments for Australian mental health care

In Australia, with growing awareness and demand for mental health services and supports, a steady increase of 3.3% in the overall perceived need for mental health care was observed among service users according to the 1997-2007 NSMHWB, especially in the areas of information, counselling, and skills training that have seen statistically significant growth in perceived need (Meadows and Bobevski, 2011). However, disparity in mental health service accessibility and delivery persisted, with no significant improvement observed in fully met need compared to partially met need (Meadows and Bobevski, 2011). Among the 13.8% of adult Australians who perceived need for mental health care in the 2007 NSMHWB, less than half reported having all their needs fully met (Meadows and Burgess, 2009). Treatment gaps were particularly notable in the areas of social intervention and skill training, with only 25.2% and 39.9% of these needs fully met, respectively, compared to higher fulfillment rates of 52.2%, 59.0%, and 84.2% for information, counselling, and medication (Meadows and Burgess, 2009).

It has been over a decade since the last NSMHWB in 2007, and with the most recent survey conducted in 2021, further research is needed to assess current levels and trends in perceived and unmet need for mental health care between 2007 and 2021. Given the evolving mental health landscape in Australia, multiple factors could contribute to the current state and development of perceived need for care:

First, mental health awareness and literacy have improved among Australians over the years, due to efforts by public campaigns and educational programs aimed at destigmatising mental health conditions, especially depression (Jorm et al., 2006a; b; Reavley and Jorm, 2011). The growing public awareness and literacy may improve individuals’ ability to recognise mental disorders, potentially increasing the perceived need for care (Jorm, 2012; Bonabi et al., 2016).

Second, increased use of medications for mental health issues has been observed among Australians in recent years (Brett et al., 2017; Australian Institute of Health and Welfare (AIHW), 2023). Since medications are generally more affordable and accessible compared to professional mental health care, an increasing number of individuals may rely on pharmacological interventions to address their mental health needs (Meadows et al., 2019). This trend may correspond to potentially higher rates of fully met medication-related needs, compared to other non-medical services, such as social or skill-based interventions and counselling.

Third, since the introduction of the Better Access Scheme in 2006, increased mental health treatment rates were observed over the years (Whiteford et al., 2014; Looi et al., 2022; Pirkis et al., 2022). This improvement in service delivery and utilisation may suggest a decrease in perceived *unmet* need, particularly for counselling services that were made more accessible through the scheme.

Fourth, economic inequity has become a prevailing issue in Australia over the decade (Australian Bureau of Statistics (ABS), 2024). With a 15% increase in household spending on goods and services based on the 2015-16 Household Expenditure Survey,^15^ the increasing costs of living may increase perceived unmet needs in the population, especially the need for social intervention such as financial and housing support. This trend is likely to disproportionately affect lower-income Australians, who may defer mental health care due to financial limitations (Enticott et al., 2016).

# Appendix C.

## Table 1. Estimates of mental health care needed in the Australian adult population (in both 2007 and 2021) including the estimates with no needs. CI, confidence interval; EPC, estimated population count; N/A, not applicable. See Figure 1 in the manuscript for a visual comparison.

| Types of help for perceived needs (demand) | | 2007 | | | | | 2021 | | | | |
| --- | --- | --- | --- | --- | --- | --- | --- | --- | --- | --- | --- |
|  |  | **People with demand** | ***Status of needs*** | | | **No demand** | **People with demand** | ***Status of needs*** | | | **No demand** |
|  |  |  | **Not met** | **Partially met** | **Fully met** |  |  | **Not met** | **Partially met** | **Fully met** |  |
| Any perceived need (demand) | EPC '000 | 2,213 | 400 | 820 | 992 | 13,780 | 3,881 | 750 | 1,256 | 1,875 | 15,144 |
|  | Overall % (95% CI) | 13.8 (13.0 - 14.7) | 2.5 (2.0 - 3.0) | 5.1 (4.5 - 5.8) | 6.2 (5.5 - 6.9) | 86.2 (85.4 - 87.0) | 20.4 (19.0 - 21.8) | 3.9 (3.2 - 4.7) | 6.6 (5.8 - 7.5) | 9.9 (8.7 - 11.0) | 79.6 (78.2 - 81.0) |
|  | % of those with need | 100% | 18.1% | 37.1% | 44.9% | N/A | 100% | 19.3% | 32.4% | 48.3% | N/A |
| Information | EPC '000 | 1,204 | 372 | 203 | 629 | 14,808 | 2,131 | 646 | 334 | 1,150 | 16,950 |
|  | Overall % (95% CI) | 7.5 (6.8 - 8.2) | 2.3 (1.9 - 2.8) | 1.3 (1.0 - 1.6) | 3.9 (3.4 - 4.5) | 92.5 (91.8 - 93.2) | 11.2 (9.9 - 12.5) | 3.4 (2.7 - 4.0) | 1.8 (1.3 - 2.2) | 6.0 (5.1 - 7.0) | 88.8 (87.6 - 90.1) |
|  | % of those with need | 100% | 30.9% | 16.9% | 52.2% | N/A | 100% | 30.3% | 15.7% | 54.0% | N/A |
| Medication | EPC '000 | 1,232 | 77 | 118 | 1,038 | 14,778 | 1,928 | 166 | 192 | 1,568 | 17,156 |
|  | Overall % (95% CI) | 7.7 (7.0 - 8.4) | 0.5 (0.3 - 0.6) | 0.7 (0.5 - 0.9) | 6.5 (5.8 - 7.2) | 92.3 (91.6 - 93.0) | 10.1 (9.0 - 11.2) | 0.9 (0.5 - 1.3) | 1.0 (0.7 - 1.4) | 8.2 (7.3 - 9.2) | 89.9 (88.8 - 91.0) |
|  | % of those with need | 100% | 6.2% | 9.6% | 84.2% | N/A | 100% | 8.7% | 10.0% | 81.4% | N/A |
| Counselling | EPC '000 | 1,695 | 437 | 258 | 1,000 | 14,319 | 3,114 | 925 | 400 | 1,789 | 15,951 |
|  | Overall % (95% CI) | 10.6 (9.9 - 11.3) | 2.7 (2.3 - 3.1) | 1.6 (1.3 - 2.0) | 6.2 (5.6 - 6.9) | 89.4 (88.7 - 90.1) | 16.3 (15.0 - 17.6) | 4.9 (4.0 - 5.7) | 2.1 (1.6 - 2.6) | 9.4 (8.2 - 10.5) | 83.7 (82.4 - 85.0) |
|  | % of those with need | 100% | 25.8% | 15.2% | 59.0% | N/A | 100% | 29.7% | 12.9% | 57.4% | N/A |
| Social intervention | EPC '000 | 670 | 433 | 68 | 169 | 15,344 | 848 | 677 | 45 | 124 | 18,223 |
|  | Overall % (95% CI) | 4.2 (3.6 - 4.8) | 2.7 (2.2 - 3.2) | 0.4 (0.2 - 0.6) | 1.1 (0.7 - 1.4) | 95.8 (95.2 - 96.4) | 4.5 (3.7 - 5.2) | 3.6 (2.8 - 4.3) | 0.2 (0.1 - 0.4) | 0.7 (0.4 - 1.0) | 95.6 (94.8 - 96.3) |
|  | % of those with need | 100% | 64.7% | 10.2% | 25.2% | N/A | 100% | 79.9% | 5.4% | 14.7% | N/A |
| Skills training | EPC '000 | 642 | 308 | 78 | 256 | 15,370 | 1,131 | 570 | 103 | 458 | 17,934 |
|  | Overall % (95% CI) | 4.0 (3.4 - 4.6) | 1.9 (0.5 - 2.3) | 0.5 (0.3 - 0.7) | 1.6 (1.2 - 2.0) | 96.0 (95.4 - 96.6) | 5.9 (5.0 - 6.8) | 3.0 (2.4 - 3.6) | 0.5 (0.3 - 0.8) | 2.4 (1.9 - 2.9) | 94.1 (93.2 - 95.0) |
|  | % of those with need | 100% | 47.9% | 12.2% | 39.9% | N/A | 100% | 50.4% | 9.1% | 40.5% | N/A |

# Appendix C. Continued

## Table 2. Estimated changes in mental health care needs among Australian adult population between 2007 and 2021. EPC, estimated population count; N/A, not applicable; ∆, change between 2021 and 2007 estimates. The comparison of demand between 2007 and 2021 was conducted using a two-proportion z-test. Significance levels: *p < 0.05, **p < 0.01, ***p<0.001.

| Types of help for perceived needs (demand) | | 2021 versus 2007 | | | | |
| --- | --- | --- | --- | --- | --- | --- |
|  |  | **People with demand** | ***Status of needs*** | | | **No demand** |
|  |  |  | **Not met** | **Partially met** | **Fully met** |  |
| Any perceived need (demand) | **∆** EPC '000 | 1,668 | 350 | 436 | 883 | 1,364 |
|  | **∆** Overall % | 6.6 (4.9, 8.3)*** | 1.4 (0.5, 2.3)** | 1.5 (0.4, 2.6)** | 3.7 (2.3, 5.1)*** | -6.6 (-8.2, -5.0)*** |
|  | **∆** % of those with need | N/A | 1.2% | -4.7% | 3.4% | N/A |
| Information | **∆** EPC '000 | 927 | 274 | 131 | 521 | 2,142 |
|  | **∆** Overall % | 3.7 (2.2, 5.2)*** | 1.1 (0.2, 2.0)* | 0.5 (-0.1, 1.1) | 2.1 (0.9, 3.3)*** | -3.7 (-5.1, -2.2)*** |
|  | **∆** % of those with need | N/A | -0.6% | -1.2% | 1.8% | N/A |
| Medication | **∆** EPC '000 | 696 | 89 | 74 | 530 | 2,378 |
|  | **∆** Overall % | 2.4 (1.1, 3.7)*** | 0.4 (0.0, 0.8) | 0.3 (-0.1, 0.7) | 1.7 (0.5, 2.9)** | -2.4 (-3.7, -1.1)*** |
|  | **∆** % of those with need | N/A | 2.5% | 0.4% | -2.8% | N/A |
| Counselling | **∆** EPC '000 | 1,419 | 488 | 142 | 789 | 1,632 |
|  | **∆** Overall % | 5.7 (4.2, 7.2)*** | 2.2 (1.2, 3.2)*** | 0.5 (-0.1, 1.1) | 3.2 (1.8, 4.6)*** | -5.7 (-7.2, -4.2)*** |
|  | **∆** % of those with need | N/A | 3.9% | -2.3% | -1.6% | N/A |
| Social intervention | **∆** EPC '000 | 178 | 244 | -23 | -45 | 2,879 |
|  | **∆** Overall % | 0.3 (-0.7, 1.3) | 0.9 (0.0, 1.8) | -0.2 (-0.5, 0.1) | -0.4 (-0.9, 0.1) | -0.2 (-1.2, 0.8) |
|  | **∆** % of those with need | N/A | 15.2% | -4.8% | -10.5% | N/A |
| Skills training | **∆** EPC '000 | 489 | 262 | 25 | 202 | 2,564 |
|  | **∆** Overall % | 1.9 (0.8, 3.0)*** | 1.1 (-0.4, 2.6) | 0.04 (-0.3, 0.4) | 0.8 (0.2, 1.4)* | -1.9 (-3.0, -0.8)*** |
|  | **∆** % of those with need | N/A | 2.5% | -3.1% | 0.6% | N/A |

# Appendix D.

## Table 3. Estimates mental health care demand (aggregate of need types) among individuals with and without a 12-month disorder (2007 and 2021): presence of disorder and broad diagnostic classes. CI, confidence interval; EPC, estimated population count. See visual comparison in Figure 2 in the main manuscript.

| 12-month mental health disorder | | 2007 | | | | | 2021 | | | | |
| --- | --- | --- | --- | --- | --- | --- | --- | --- | --- | --- | --- |
|  |  | **People with demand** | ***Status of needs*** | | | **No demand** | **People with demand** | ***Status of needs*** | | | **No demand** |
|  |  |  | **Not met** | **Partially met** | **Fully met** |  |  | **Not met** | **Partially met** | **Fully met** |  |
| No 12-month mental health disorder | EPC '000 | 827 | 94 | 254 | 479 | 11,987 | 1,471 | 212 | 330 | 927 | 13,461 |
|  | Overall % (95% CI) | 6.5 (5.7 - 7.2) | 0.7 (0.5 - 1.0) | 2.0 (1.6 - 2.4) | 3.7 (3.1 - 4.4) | 93.5 (92.1 - 95.0) | 9.9 (8.2 - 11.6) | 1.4 (0.9 - 1.9) | 2.2 (1.5 - 2.9) | 6.2 (4.9 - 7.5) | 90.1 (88.37 - 91.83) |
|  | % of those with need | 100% | 11.4% | 30.7% | 57.9% | N/A | 100.0% | 14.4% | 22.5% | 63.1% | N/A |
| Any 12-month mental health disorder | EPC '000 | 1,385 | 306 | 566 | 513 | 1,812 | 2,410 | 537 | 925 | 947 | 1,682 |
|  | Overall % (95% CI) | 43.3 (39.8 - 46.8) | 9.6 (7.6 - 11.5) | 17.7 (15.0 - 20.4) | 16.0 (13.9 - 18.2) | 56.7 (51.9 - 61.5) | 58.9 (55.1 - 62.7) | 13.1 (10.1 - 16.2) | 22.6 (19.8 - 25.4) | 23.1 (19.2 - 27.0) | 41.1 (37.29 - 44.91) |
|  | % of those with need | 100% | 22.1% | 40.9% | 37.0% | N/A | 100.0% | 22.3% | 38.4% | 39.3% | N/A |
| Any 12-month affective disorder | EPC '000 | 698 | 132 | 326 | 240 | 298 | 1,104 | 245 | 437 | 420 | 350 |
|  | Overall % (95% CI) | 70.1 (60.7 - 79.5) | 13.2 (8.8 - 17.6) | 32.7 (25.6 - 39.9) | 24.1 (19.2 - 29.1) | 29.9 (22.9 - 36.9) | 75.9 (70.5 - 81.3) | 16.9 (11.1 - 22.7) | 30.1 (24.4 - 35.8) | 29.0 (23.1 - 34.9) | 24.1 (18.72 - 29.48) |
|  | % of those with need | 100% | 18.9% | 46.7% | 34.4% | N/A | 100.0% | 22.3% | 39.6% | 38.1% | N/A |
| Any 12-month substance use disorder | EPC '000 | 259 | 65 | 107 | 87 | 561 | 267 | 53 | 150 | 63 | 352 |
|  | Overall % (95% CI) | 31.6 (23.9 - 39.2) | 8.0 (4.6 - 11.4) | 13.0 (7.9 - 18.1) | 10.6 (5.5 - 15.7) | 68.4 (57.7 - 70.2) | 43.1 (31.3 - 54.9) | 8.6 (2.8 - 14.4) | 24.4 (13.2 - 35.6) | 10.1 (3.8 - 16.4) | 56.9 (45.06 - 68.74) |
|  | % of those with need | 100% | 25.2% | 41.2% | 33.6% | N/A | 100.0% | 20.0% | 56.5% | 23.5% | N/A |
| Any 12-month anxiety disorder | EPC '000 | 1,057 | 215 | 463 | 379 | 1,246 | 1,965 | 421 | 802 | 741 | 1,245 |
|  | Overall % (95% CI) | 45.9 (41.1 - 50.7) | 9.3 (7.1 - 11.5) | 20.1 (16.5 - 23.6) | 16.5 (14.0 - 19.0) | 54.1 (48.5-59.7) | 61.2 (56.8 - 65.6) | 13.1 (9.8 - 16.4) | 25.0 (21.4 - 28.6) | 23.1 (18.9 - 27.3) | 38.8 (34.4 - 43.2) |
|  | % of those with need | 100% | 20.3% | 43.8% | 35.9% | N/A | 100.0% | 21.4% | 40.8% | 37.7% | N/A |

# Appendix D. Continued

## Table 4. Estimated changes in mental health care demand (aggregate of need types) among individuals with and without a 12-month disorder between 2007 and 2021. EPC, estimated population count; N/A, not applicable; ∆, change between 2021 and 2007 estimates. The comparison of demand between 2007 and 2021 was conducted using a two-proportion z-test. Significance levels: *p < 0.05, **p < 0.01, ***p<0.001.

| 12-month mental health disorder | | 12-month mental health disorder | | | | |
| --- | --- | --- | --- | --- | --- | --- |
|  |  | **People with demand** | ***Status of needs*** | | | **No demand** |
|  |  |  | **Not met** | **Partially met** | **Fully met** |  |
| No 12-month mental health disorder | **∆** EPC '000 | 644 | 118 | 77 | 448 | 1,474 |
|  | **∆** Overall % | 3.4 (1.5, 5.3)*** | 0.7 (0.1, 1.3)* | 0.2 (-0.6, 1.0) | 2.5 (1.0, 4.0)*** | -3.4 (-5.7, -1.1)** |
|  | **∆** % of those with need | N/A | 3.0% | -8.2% | 5.2% | N/A |
| Any 12-month mental health disorder | **∆** EPC '000 | 1,025 | 231 | 359 | 434 | -130 |
|  | **∆** Overall % | 15.6 (10.4, 20.8)*** | 3.5 (-0.2, 7.2) | 4.9 (1.0, 8.8)* | 7.1 (2.6, 11.6)** | -15.6 (-21.7, -9.5)*** |
|  | **∆** % of those with need | N/A | 0.2% | -2.5% | 2.3% | N/A |
| Any 12-month affective disorder | **∆** EPC '000 | 406 | 113 | 111 | 180 | 52 |
|  | **∆** Overall % | 5.8 (-5.0, 16.6) | 3.7 (-3.6, 11.0) | -2.6 (-11.8, 6.6) | 4.9 (-2.8, 12.6) | -5.8 (-14.6, 3.0) |
|  | **∆** % of those with need | N/A | 3.4% | -7.1% | 3.7% | N/A |
| Any 12-month substance use disorder | **∆** EPC '000 | 8 | -12 | 43 | -24 | -209 |
|  | **∆** Overall % | 11.5 (-2.6, 25.6) | 0.6 (-6.1, 7.3) | 11.4 (-0.9, 23.7) | -0.5 (-8.6, 7.6) | -11.5 (-27.5, 4.5) |
|  | **∆** % of those with need | N/A | -5.2% | 15.3% | -10.1% | N/A |
| Any 12-month anxiety disorder | **∆** EPC '000 | 908 | 206 | 339 | 362 | -1 |
|  | **∆** Overall % | 15.3 (8.8, 21.8)*** | 3.8 (-0.2, 7.8) | 4.9 (-0.2, 10.0) | 6.6 (1.7, 11.5)** | -15.3 (-22.4, -8.2)*** |
|  | **∆** % of those with need | N/A | 1.1% | -3.0% | 1.8% | N/A |

# References:

Australian Bureau of Statistics (ABS) (2024) *Income and wealth inequality - Dynamic economy that shares prosperity [Internet]*. Available at: <https://www.abs.gov.au/statistics/measuring-what-matters/measuring-what-matters-themes-and-indicators/prosperous/income-and-wealth-inequality> (accessed 2024 Oct 1).

Australian Institute of Health and Welfare (AIHW) (2023) *Mental health-related prescriptions [Internet]*. Available at: <https://www.aihw.gov.au/mental-health/topic-areas/mental-health-prescriptions> (accessed 2023 Nov 2).

Bonabi H, Müller M, Ajdacic-Gross V, et al. (2016) Mental Health Literacy, Attitudes to Help Seeking, and Perceived Need as Predictors of Mental Health Service Use: A Longitudinal Study. *J Nerv Ment Dis* 204(4): 321-324.

Brett J, Karanges EA, Daniels B, et al. (2017) Psychotropic medication use in Australia, 2007 to 2015: Changes in annual incidence, prevalence and treatment exposure. *Australian and New Zealand Journal of Psychiatry* 51(10): 990-999.

Enticott JC, Meadows GN, Shawyer F, et al. (2016) Mental disorders and distress: Associations with demographics, remoteness and socioeconomic deprivation of area of residence across Australia. *Australian and New Zealand Journal of Psychiatry* 50(12): 1169-1179.

Jorm AF (2012) Mental health literacy: empowering the community to take action for better mental health. *Am Psychol* 67(3): 231-243.

Jorm AF, Christensen H and Griffiths KM (2006a) Changes in Depression Awareness and Attitudes in Australia: The Impact of Beyondblue: The National Depression Initiative. *Australian & New Zealand Journal of Psychiatry* 40(1): 42-46.

Jorm AF, Christensen H and Griffiths KM (2006b) The Public's Ability to Recognize Mental Disorders and their Beliefs about Treatment: Changes in Australia Over 8 Years. *Australian & New Zealand Journal of Psychiatry* 40(1): 36-41.

Looi JCL, Maguire PA, Allison S, et al. (2022) Medicare-subsidised mental health services from the beginning of Better Access in 2006–2007 to 2019–2020: Descriptive analysis by state, profession and consultation profile. *Australasian Psychiatry* 30(5): 640-652.

Meadows GN and Bobevski I (2011) Changes in met perceived need for mental healthcare in Australia from 1997 to 2007. *British Journal of Psychiatry* 199(6): 479-484.

Meadows GN and Burgess PM (2009) Perceived need for mental health care: Findings from the 2007 Australian Survey of Mental Health and Wellbeing. *Australian and New Zealand Journal of Psychiatry* 43(7): 624-634.

Meadows GN, Prodan A, Patten S, et al. (2019) Resolving the paradox of increased mental health expenditure and stable prevalence. *Australian & New Zealand Journal of Psychiatry* 53(9): 844-850.

Pirkis J, Currier D, Harris M, et al. (2022) Evaluation of the Better Access initiative. Reportno. Report Number|, Date. Place Published|: Institution|.

Reavley NJ and Jorm AF (2011) Recognition of Mental Disorders and Beliefs about Treatment and Outcome: Findings from an Australian National Survey of Mental Health Literacy and Stigma. *Australian & New Zealand Journal of Psychiatry* 45(11): 947-956.

Whiteford HA, Buckingham WJ, Harris MG, et al. (2014) Estimating treatment rates for mental disorders in Australia. *Australian Health Review* 38(1): 80-85.
